# Two-year outcome after recurrent first trimester miscarriages: prognostic value of the past obstetric history

**DOI:** 10.1007/s00404-015-4001-x

**Published:** 2016-01-21

**Authors:** Christiane Kling, Julia Magez, Jürgen Hedderich, Sören von Otte, Dieter Kabelitz

**Affiliations:** Institute of Immunology, University Hospital Schleswig–Holstein, Campus Kiel, Arnold-Heller-Str. 3 Haus 17, 24105 Kiel, Germany; Institute of Medical Statistics and Informatics, University Hospital Schleswig–Holstein, Campus Kiel, Arnold-Heller-Str. 3 Haus 17, 24105 Kiel, Germany; Fertility Centre, University Hospital Schleswig–Holstein, Campus Kiel, Arnold-Heller-Str. 3 Haus 17, 24105 Kiel, Germany; Department of General Practice and Health Services Research, University Hospital, Heidelberg, Germany

**Keywords:** Spontaneous abortion, Pregnancy loss, Embryo, Cardiac activity, Infertility, Karyotype

## Abstract

**Purpose:**

Recurrent miscarriage (RM) is a stressful condition which gives rise to extensive diagnostic evaluation and is seen as a potentially curable maternal disease. Nevertheless, epidemiological data have shown that outcome is related to fertility. In addition to maternal age and number of preceding miscarriages, further markers derived from the past history may support counselling.

**Methods:**

Observational trial comprising 228 couples who were referred between 1996 and 2003 for immunological evaluation at maternal ages 20–39 years after three or more spontaneously conceived primary first trimester miscarriages. They were interviewed in 2005, ongoing pregnancies were followed up until birth in 2006. Past obstetric history was correlated with 2 year cumulative pregnancy and delivery rates (CPR, CDR).

**Results:**

CPR and CDR were 206/228 (90.4 %) and 174/228 (76.4 %). Duration of infertility was associated with lower CPR (up to 3/>3 years, *p* < 0.01), whereas age and number of preceding losses inversely correlated with CDR (<35 years/35–39 years, *p* < 0.002; 3/>3 miscarriages, *p* < 0.002). Detection of an embryonic heart beat in 2–3 of the first three miscarriages resulted in favourable outcome (CPR: *p* < 0.02, CDR: *p* < 0.002). Prognosis was excellent in younger fertile women after three miscarriages where vital signs had been detected; under less favourable conditions not only risks for further miscarriage, but also for secondary infertility were elevated.

**Conclusion:**

Secondary infertility is a feature of RM. Embryonic vital signs in preceding pregnancies are prognostic markers and should be regarded as a strong confounding factor in trials on therapeutic interventions. Prevention may be more appropriate than treatment.

## Introduction

About 12–15 % of clinical pregnancies end in spontaneous abortion, and an estimated 1 % of women who desire children suffer from recurrent miscarriages. According to the WHO, this term is defined as consecutive loss of three or more pregnancies up to the 20th gestational week [1], and 90 % occur in first trimester [2]. International guidelines of the gynaecological societies adopted the WHO definition, and from the knowledge that about 50 % of losses are caused by cytogenetic abnormalities while 50 % are chromosomally normal, they followed the concept that euploid losses indicate a maternal and potentially curable disease.

Although embryonic factors which lead to aneuploidy are the only clearly defined cause of miscarriage, karyotypic evaluation of the abortus has not become a routine procedure due to technical and financial reasons. Therefore, the guidelines concentrate on evaluation of associated maternal factors deducing therapeutic options [3–5]. Nevertheless, about 25–50 % of cases remain unexplained according to this model [5, 6], and cure rates have been stable for the past 60 years ranging from 55 to 85 % [7–10].

In parallel, epidemiological evidence has shown that the prospects after recurrent miscarriages can be excellent. Moreover, factors derived from the obstetric history, e.g., maternal age and number of preceding miscarriages, independently have a negative impact on the prognosis [8, 11, 12]. Miscarriages have also been linked to subfertility [13].

In an observational follow-up study, we intended to get a basis for counselling couples on their prospects within the near future. Basic information derived from the past history was correlated not only with the chance to conceive a child but also to get pregnant at all. We concentrated on a group of women below 40 years with primary miscarriages who had conceived spontaneously. Morphological examination by ultrasound has revealed that in sporadic pregnancies, the establishment of cardiac activity is an important milestone of embryonic organogenesis. The chance of a pregnancy to develop normally is markedly higher after embryonic vital sign have been detected. For women below 35–40 years, the figures rise from 75 to 90–95 % [14–16]. Since this parameter is routinely examined in early pregnancy as a part of prenatal care, we evaluated whether it is also prognostically relevant in case of pregnancy loss.

We did not analyse the possible effect of secondary treatments, e.g., lymphocyte immunotherapy, ASS or low molecular weight heparin because we did not expect them to “override” biological factors which are derived from the obstetric history and which are related to the capacity of the early embryo to develop. The results may contribute to an extended approach of guiding couples who suffer from miscarriages.

## Materials and methods

### Academic centre

After three or more miscarriages, couples are referred to our tertiary immunological practice by gynaecologic practitioners, geneticists, and reproductive medical centres in Germany. Lymphocyte immunotherapy (LIT) is considered as an optional treatment in selected cases of primary idiopathic recurrent miscarriages as defined by the gynaecological guidelines. The method has been introduced in our centre in the 1980s and is approved by the local health authority. The criteria for recommending LIT have been outlined elsewhere. Surveillance after LIT showed very low or undetectable risks for the woman treated and the children born subsequently [17, 18].

### Study design

Observational study on 336 couples who were consecutively referred between 1996 and 2003 and fulfilled the following criteria: three or more miscarriages from the 5th to 15th gestational week after spontaneous conception, female age below 40 years, no deliveries, no late miscarriages, no pregnancy in a preceding partnership. The diagnostic work-up by the referring practitioners partly adhered to the contemporary guidelines for diagnosis and treatment and comprised pelvic ultrasound (all cases), TSH (all cases), hysteroscopy (103 women, 45.2 %), cytogenetic evaluation of both partners (195 couples, 85.5 % revealing normal results), haemostaseologic evaluation (complete numbers tested uncertain), and details concerning the past miscarriages (gestational age, detection of embryonic vital signs, embryonic aneuploidy were tested). Fifty-eight (25.4 %) women were tested for autoantibodies and antiphospholipid syndrome. Missing or abnormal diagnostic results did not lead to exclusion from the study [19].

In 2005, they were contacted a second time. Of 228 eligible couples (67.9 %), 144 returned the questionnaire, 222 were contacted by telephone, and in 30 cases additional information was given by the practitioners with the patients’ consent. Time of observation was 24–108 months after initial immunological evaluation (*n* = 114) and after LIT (*n* = 114), respectively. Pregnancies were followed up until delivery in 2006. Within a 24 month period, mean outcome measures were the cumulative pregnancy and birth rate, meaning the rate of conceptions leading to first delivery.

In the cohort, 114 (50 %) had received LIT, the others had no met the LIT criteria (e.g., other causes for recurrent miscarriages detected, no cytogenetic workup, HLA antibodies present, partner not suited for cell donation according to the guidelines of donor selection in transfusion medicine).

Of 336 couples, 84 (25 %) were lost to follow up, and 24 (7.1 %) turned out not to fulfil the criteria of the trial (*n* = 11) or had stopped trying to achieve pregnancy (*n* = 13). The loss of follow up rate was higher in couples who had their initial evaluation before 1999 and who did not undergo LIT. It is partly explained by loss of the current addresses. The concept of the study was approved by the ethics committee of the medical faculty of Kiel University and by the local data protection official.

### Definition of gestational age

In Germany, prenatal care of gynaecological practitioners includes an ultrasound scan in gestational weeks 8+0 to 11+6 in order to detect gross malformations and to define the prospective date of delivery. Moreover, ultrasound scans are widely used to detect an intact intrauterine pregnancy in the 6th–8th gestational week. Gestational age in very early pregnancy is defined as weeks after the last menstrual period, from the 7th week on it is based on the ultrasound findings. The embryonic phase of development is completed in the 10th week, and developmental arrest in this phase becomes apparent until the 12th week.

### Statistics

Study data are described by proportions and rates as well as by mean and median values (ranges, standard deviations) for quantitative variables. Stepwise multiple logistic regression analysis was used to assess the influence of selected factors from the past obstetric history on CPR and CDR. In this exploratory study a *p* value <0.05 was considered significant. The effect of selected markers was evaluated by odds ratios incl. 95 % confidence limits.

## Results

### Study group

**Fig. 1:**
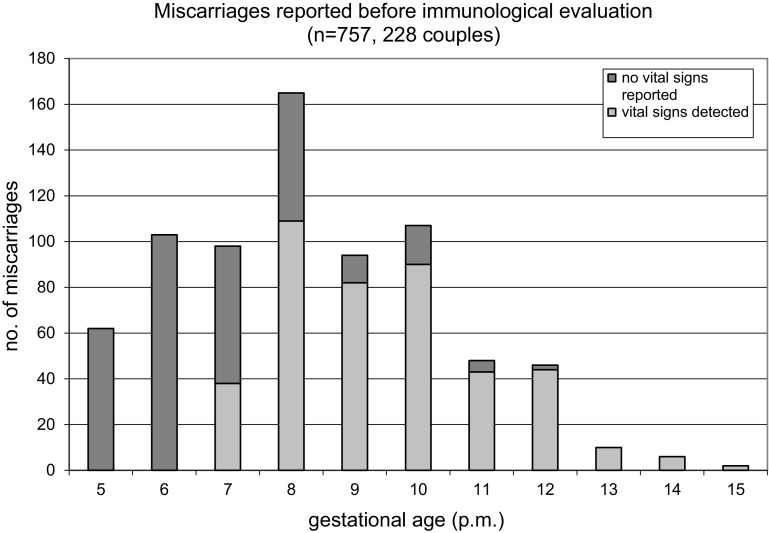
Miscarriages reported before immunological evaluation (*n* = 757, 228 couples)

A group of 228 couples underwent initial immunological evaluation (IE) in 1996–2003 after at least three first trimester miscarriages and gave sufficient information in 2005. At IE the women were 32 years old (median, 21–39 years) and had 3 miscarriages (median, range 3–7) within a waiting time of 3 (0–16) years: 171 women (75.0 %) had three, 43 (18.9 %) four, 11 (4.8 %) five, 2 (0.9 %) six, and 1 couple (0.4 %) had 7 miscarriages. Mean gestational age at time of miscarriages was 8.4 ± 1.1 weeks (5–15 weeks), in 431 of 759 miscarriages (56.8 %) the embryonic heart beat was detected from the 7th week on, with close similarity in all maternal age groups (Table 1; Fig. 1).

**Table 1:** Past obstetric history and outcome after immunological evaluation (IE)

Female age at IE	21–29 years	30–34 years	35–39 years	Total
No.of couples	66	106	56	228
Median duration of infertility (years, range)	2 (0–7)	3 (0–7)	3.5 (0–16)	3 (0–16)
Miscarriages before IE:				
Three	52 (78.8 %)	80 (75.5 %)	39 (69.6 %)	171 (75.0 %)
Four	12 (18.2 %)	18 (17.0 %)	13 (23.2 %)	43 (18.9 %)
5–7	2 (3.0 %)	8 (7.5 %)	4 (7.1 %)	14 (6.1 %)
Total no. of pregnancies	216	357	193	766
Of these extrauterine	0	5	2	7
Of these intrauterine	216	352	191	759
Median no. per couples (range)	3 (3–7)	3 (3–5)	3 (3–6)	3 (3–7)
Vital signs present	125 (57.8 %)	192 (54.5 %)	109 (57.1 %)	426 (55.6 %)
Biochemical/preclinical (4th–5th week)	18 (8.3 %)	33 (9.4 %)	11 (5.8 %)	62 (8.2 %)
Embryonic phase (6th–12th week)	192 (88.9 %)	312 (88.6 %)	173 (90.6 %)	677 (89.2 %)
Early foetal phase (13th–15th week)	7 (3.2 %)	7 (2.0 %)	5 (2.6 %)	19 (2.5 %)
Mean interpregnancy interval (months, range)	9.3 ± 3.5 (4–30)	9.1 ± 3.6 (2.5–30)	10.9 ± 4.7 (3.5–33.5)	9.8 ± 3.9 (2.5–33.5)
Outcome 2 years after IE				
Couples with further pregnancies	60 (90.9 %)	100 (94.3 %) (of these 1 extrauterine)	46 (82.1 %)	206 (90.4 %)
Of these:				
Conception of first child	51 (77.3 %)	90 (84.9 %)	33 (58.9 %)	174 (76.4 %)
Miscarriages only	9 (13.6 %)	10 (9.4 %) (of these 1 extrauterine)	13 (23.2 %)	32 (14.0 %)
Couples with secondary infertility	6 (9.1 %)	6 (5.7 %)	10 (17.9 %)	22 (9.6 %)
Total no. of pregnancies	78	128	55	261
Pregnancies per couple (range)	1.18 (0–3)	1.21 (0–3)	0.98 (0–2)	1.14 (0–3)
Male factor infertility	8 (12.1 %)	8 (7.5 %)	10 (17.9 %)	26 (11.4 %)
Tubal impairment	3 (4.5 %)	12 (11.3 %)	5 (8.9 %)	20 (8.9 %)

Within 24 months after IE, 206 woman achieved pregnancy (90.4 %), and 22 (9.6 %) developed secondary infertility. 174 women (76.4 %) conceived their first child, 10 of them their second one. Thirty-four of 228 (14.9 %) commenced infertility treatment using allogeneic insemination with the husband’s sperm (AIH) or in vitro fertilisation (IVF). Women under 30 years reported on this most frequently (12/66, 18.2 %). With infertility treatment, 12 of 34 women were delivered, and 4 finally conceived spontaneously. Of 12 pregnancies conceived after treatment, five ended prematurely, and three of these very prematurely.

### Evaluation of associated pathologies of recurrent miscarriages

Andrological impairment of variable extent was found in 26 of 141 partners, and 80 (35.1 %) of the female patients reported on abnormal findings: tubal incompetence (extrauterine pregnancy ×6, unilaterial salpingectomy ×4, adhesions ×3), uterine (myoma ×4, septate uterus ×5, polyp ×1, conisation for cervical dysplasia ×4), endometriosis (*n* = 5), thyroid disease (Hashimoto thyroiditis, struma, *n* = 11), endocrine (corpus luteum incompetence, polycystic ovary syndrome, hyperandrogenaemia, hyperprolactinaemia, *n* = 28), factor V Leiden mutation (*n* = 9). Cytogenetic aneuploidy of the embryo was found in 16 women.

### Cumulative pregnancy and delivery rates within 24 months

Dichotome variables derived from the past obstetric history were related to the cumulative pregnancy and delivery rates: female age (20–34 years/35–39 years), partner’s age (20–34 years/>34 years), number of miscarriages (3/>3), duration of infertility (1–3/>3 years), and embryonic vital signs in the first three miscarriages (0–1/2–3). While the age of the male partner did not have a significant impact, the pregnancy rate was significantly higher when the waiting time was less than 3 years, when vital signs had been present in most of the first three miscarriages, and there was a trend concerning the number of preceding miscarriages. The conception rate was higher in younger women (below 35 years), with low numbers of miscarriages and detection of embryonic vital signs (Table 2).

**Table 2:** Multivariate logistic regression on prognostic factors and cumulative pregnancy and delivery rates 2 years after immunological evaluation (IE) (228 couples)

Prognostic parameters	Dichotome categories	Couples achieving pregnancies total: 206/228 (90.4 %)	Odd’s ratio	*P* value	Confidence interval	Conception of first child total: 174/228 (76.4 %)	Odd’s ratio	*P* value	Confidence interval
Female age at IE	20–34 years	160/172 (93.0 %)	0.41	0.073 n.s.	0.16–1.09	141/172 (82.0 %)	**0.28**	**0.001**	0.14–0.58
	35–39 years	46/56 (82.1 %)				34/56 (60.7 %)			
Age of male partner at IE	21–34 years	130/141 (92.2 %)	1.06	0.924 n.s.	0.35–3.22	115/141 (81.6 %)	0.71	0.361 n.s.	0.34–1.49
	>34 years	76/67 (87.4 %)				59/67 (67.8 %)			
Duration of infertility	0–3 years	144/153 (94.1 %)	**0.27**	**0.006**	0.11–0.69	124/153 (81.0 %)	0.57	0.120 n.s.	0.28–1.16
	>3 years	62/75 (82.7 %)				51/75 (68.0 %)			
No. of preceding miscarriages	3	159/171 (93.0 %)	0.40	0.058* n.s.	0.16–1.03	141/171 (82.5 %)	**0.33**	**0.002**	0.16–0.67
	>3	47/57 (82.5 %)				34/57 (59.6 %)			
Embryonic heart beat in the first 3 pregnancies detected	0–1	88/103 (85.4 %)	**3.35**	**0.016**	1.26–8.92	67/103 (65.0 %)	**3.25**	**0.001**	1.64–6.43
	2–3	118/125 (94.4 %)				108/125 (86.4 %)			

**Fig. 2:**
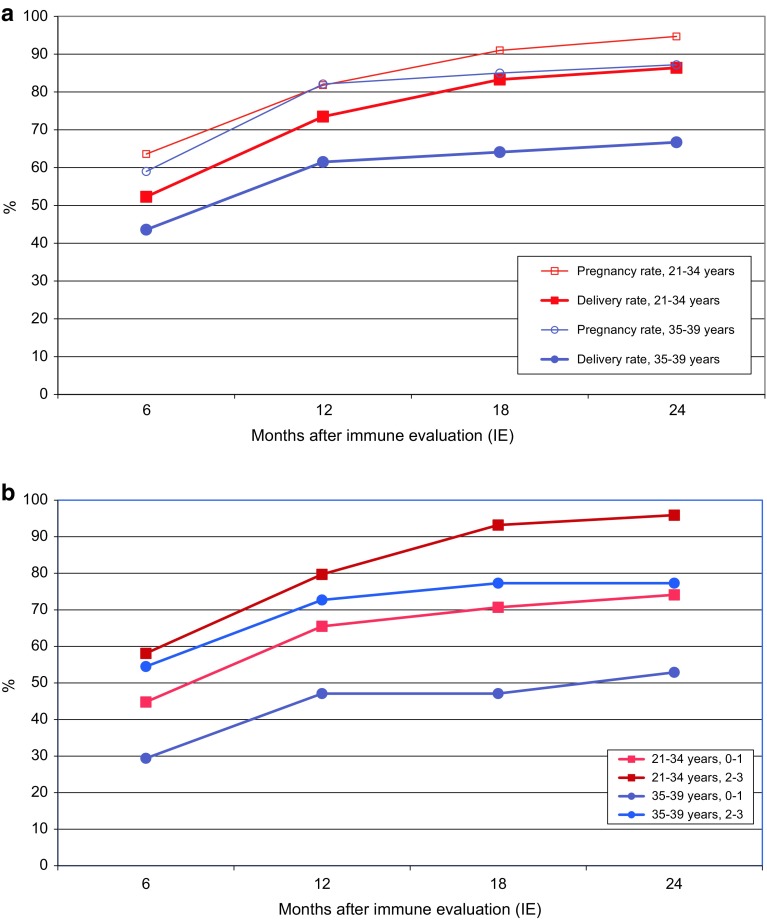
**a** Cumulative pregnancy and delivery rates after three early miscarriages, dependant on female age (*n* = 171). **b** Cumulative delivery rates after three early miscarriages, dependant on female age and detection rate of vital signs in preceding miscarriages (*n* = 171)

The main group of 132 women below 35 years had an overall chance of 85 % to conceive their first child after three miscarriages, and the rate was nearly 100 %, when embryonic vital signs had been present before. Under least favourable conditions the pregnancy rate dropped to 70 % and the delivery rate to 40 % (Fig. 2a, b; Table 3).

**Table 3:** Cumulative pregnancy and delivery rates related to criteria derived from the past obstetric history

Categories	Age below 35 years	Three miscarriages only	Embryonic heart beat in 2-3 of the first three miscarriages detected	Patients total: 228	Patients achieving pregnancy within 2 years total: 206	Cumulative pregnancy rate (CPR)(%) total: 90.3%	Patients conceiving their first child within 2 years total: 174	Cumulative delivery rate (CDR) (%) Total: 76.3 %
1	No	No	No	7	5	71.4	3	42.9
2	No	No	Yes	10	7	70.0	4	40.0
3	No	Yes	No	17	15	88.2	9	52.9
4	Yes	No	No	21	17	81.0	13	61.9
5	No	Yes	Yes	22	19	83.4	17	77.3
6	Yes	No	Yes	19	18	94.7	14	73.7
7	Yes	Yes	No	58	51	87.9	43	74.1
8	Yes	Yes	Yes	74	74	100	71	95.9

The categories are based on the odd’s ratios (Table 2) of the defined criteria which are significantly correlated with the observed outcome. When 47 couples are excluded because of reported sterility factors (tubal incompetence, uterine disorders, andrological), figures do not change significantly: 168/181 couples (92.8 %) achieved pregnancy (*p* = 0.38), 145/181 couples (80.1 %) were delivered (*p* = 0.38). CPR: 75 %/66.7 %/88.9 %/88.2 %/83.3 %/94.4 %/95.1 %/100 % in the six categories, CDR: 25 %/44.4 %/55.6 %/70.6 %/77.8 %/72.2 %/80.5 %/96.9 %

Possible factors indicating reduced fertility (reduced sperm count, tubal pathology, preceding extrauterine pregnancies, and uterine abnormalities, 47 couples) were not significantly associated with the outcome at univariate analysis (data not shown).

### Deliveries after recurrent miscarriages

Of 174 women conceiving their first child, 170 had singleton pregnancies (89 girls, 81 boys) and 4 had multiples following infertility treatment: three pairs of twins (3 girls, 3 boys), and one triplet with only one boy developing to term.

The rate of premature deliveries (24th to completed 37th gestational week) was 16.7 % (29/174), and the rate of very premature deliveries was 2.9 % (5/174, 24th to 31st week). In 83 cases (47.7 %), complications of pregnancy were reported, the most frequent being vaginal bleeding in the first trimester (*n* = 45, Table 4). Two very premature infants succumbed pre- and one postnatally (3 of 177 infants, 1.7 %). Eleven of 174 living infants (6.3 %) had major malformations or handicaps. These comprised trisomy 21, cardiac anomaly, Jeune’s syndrome, spinal muscular atrophy, unilateral renal agenesis, hypospadia, connatal deafness, and neuromuscular retardation (*n* = 4). The rate of these impairments was 3 of 51 (6 %) in women aged 21–29 years, 3 of 90 (3 %) in women aged 30–34 years, and remarkably high (5 of 33, 15 %) in those aged 35–39 years, as compared to the younger women (*p* = 0.02). One of these 5 children was born after embryo transfer to a mother aged 37 years, the others were conceived spontaneously.

**Table 4:** Prenatal complications (83 of 174 women, 47.4 %)

Complication	No. of women (%)
First trimester bleeding	45 (25.9)
Placenta praevia	1 (0.6)
Premature placental abruption	2 (1.1)
Placenta accreta	3 (1.7)
Perinatal demise	3 (1.7)
Pre-eclampsia, hypertension	7 (4.0)
Gestational diabetes	10 (5.7)
Acute or chronic placental incompetence, intrauterine retardation	9 (5.2)
Cervical incompetence	8 (4.6)
Premature rupture of membranes (ROM)	4 (2.3)
Premature labour	6 (3.4)
Pyelonephritis	1 (0.6)
Infection (amnion, cervix)	8 (4.6)
Pulmonary embolism	1 (0.6)

## Discussion

Our data illustrate that after three early miscarriages the chance to conceive the first child within 2 years is good or excellent for younger women who have achieved their first pregnancies spontaneously. They also confirm current knowledge that the chances decrease with the number of miscarriages as well as with female age because of a higher risk of further miscarriage. As we could show here, secondary sterility accounts for the decrease as well and therefore is a characteristic feature of recurrent miscarriages.

Additionally, our data provide evidence that vital signs found in preceding pregnancies can serve as a non-invasive prognostic marker in recurrent miscarriages. When the preceding embryos have reached a higher developmental age, the chances to get pregnant as well as to conceive successfully are significantly higher than after repeated early disruption. We conclude that sporadic and even more so recurrent early miscarriages do not simply occur accidentally, but reflect the intrinsic fertility of a woman. The role of her partner is difficult to define to date. At least will reduced male fertility account for longer waiting times between pregnancies.

As a technique requiring an invasive procedure, cytogenetic evaluation of the conceptus obtained by uterine evacuation or by chorionic villi sampling has been established since the 1960s and late 1980s, respectively. Depending on gestational and female age, about 50–80 % of early miscarriages are caused by aneuploidy of the embryo or—much less frequently proven—to mosaicism which may be confined to the placenta only [20–22]. The patterns of aneuploidy differ with maternal and gestational ages, and the type of aneuploidy roughly determines the developmental age the conceptus is able to reach. Thus, nonviable trisomies 16 or 22 can present as anembryonic structures or proceed to cardiac activity [23, 24]. As has been shown in detail for monosomy X, the whole spectrum from a “blighted ovum” to delivery of a viable child may be possible [25]. While monosomic embryos are usually unable to implant, not only monosomy X, but also triploidy, and certain trisomies (e.g., trisomies 13, 18, 21, triple X, XXY) allow for organogenesis and even viability. At the same time, these trisomies are more prevalent in elder women. Nevertheless, the conclusion that vital signs are more often detected in elder women cannot be drawn from our collective (Table 1).

From the geneticist´s point of view, the developmental stage which can be reached in aneuploidy is explained by the degree of genetic imbalance caused by extra or missing chromosomes. Nevertheless, it is unclear which factors actually lead to developmental arrest. Structural abnormalities do not only occur in aneuploid, but also in chromosomally normal embryos [26, 27]. After missed abortion, Phillip found that only 7 % had a normal karyotype and were morphologically intact [28]. This has been attributed to monogenic lethal factors [29]. There may be additional factors originating from the maturation of the oocyte and the constitution of the embryo which influence its capacity to develop; e.g., at early cleavage stages, the embryo apparently is able to overcome mosaicism [21]. These factors may explain why vital signs in first trimester pregnancy indicate a favourable prognosis although it may be aneuploid.

An additional impact of environmental factors on early embryonic development and nidation, e.g., the tubal and endometrial milieu cannot be excluded, and tubal impairment, uterine malformations, septum, or submucous myoma may have detrimental effects. Nevertheless, they are frequently observed, and their role in recurrent miscarriages is discussed controversially to date [3, 4, 30]. Moreover, together with andrological subfertility they can contribute to secondary sterility. In our collective, required diagnostic evaluation and infertility treatment may have delayed pregnancy in some couples within 24 months of observation. Remarkably though, after exclusion of all 47 couples who had reported on andrological, tubal, or uterine impairment, success rates of 181 remaining couples were merely unchanged (see Table 3).

Our results lead to the conclusion that the chance to detect vital signs decrease with higher grade recurrent miscarriages due to selection. This has also been observed but to our knowledge has not been studied specifically by other authors [11, 31]. In order to avoid a bias, we chose to weigh only the first three miscarriages. Further studies are necessary to rule out whether the marker is equally valid in higher grade recurrent miscarriages or in women age 40 years and older. Nevertheless, it has a strong impact in women under 40 years of age and should be regarded as a strong confounding factor whenever diagnostic or therapeutic measures for couples suffering from recurrent miscarriages are evaluated. We speculate that in a population with miscarriages following fertility treatment, the chance to detect vital signs will be lower. Therefore it may be relevant to describe whether a study population comprises couples requiring fertility treatment.

By interviewing the patients closely, we obtained detailed clinical information on past history and further outcome. Although the evaluation has been done in 2005 and refers to a collective who had been seen with recurrent miscarriages as far as 9 years earlier, we think that the results are still valid because all women had conceived spontaneously. Since the loss of follow-up rate (25 %) is attributed to loss of current addresses we presume that the results are not markedly distorted. They are plausible when compared to other studies (as below, Table 4).

Concerning diagnostic results, the mode of obtaining data by reports of the referring practitioners and the patients may be a weakness of our study. The ultrasound and gynaecological evaluation were performed by the referring practitioners and not subject to standardised study criteria. In case of missed abortion the developmental age of the embryo had not been defined. We cannot exclude that in some 7th week losses where no heart beat has been reported, ultrasound had not actually taken place. Nevertheless, information on this aspect has been obtained at least twice (at immunological evaluation, at LIT, and finally by means of a questionnaire), and seems plausible (Fig. 1).

Our collective does not represent a group of idiopathic recurrent miscarriages in its classical sense as women have not been excluded on the basis of pathological or missing genetic, gynaecologic or laboratory results. At present knowledge, an inherited heterozygous mutation of coagulation factors (e.g., factor V Leiden) or detection of antiphospholipid antibodies are not clinically relevant in first trimester abortions but rather may underlie pathologies in the second or third trimester [32–35]. Therefore, lack of the respective laboratory results is not regarded as a confounding factor. Nearly 15 % of the couples have not been evaluated cytogenetically. Since the detection rate after recurrent early loss is about 2–5 % [4], this factor is considered negligible in our cohort. It would have been valuable to stratify for hormonal impairment like polycystic ovary syndrome, female body mass index, or life style factors such as nicotine and alcohol consumption, but these aspects were not included at the primary assessment of the data.

In our evaluation, unexpectedly a significant impact of female age on the cumulative pregnancy rate could not be shown. Two reasons may have contributed: women in their 40s were not included, and women aged 30–34 years possibly decided more easily for another pregnancy than younger women. Moreover, a higher proportion of the young couples apparently had underlying fertility problems. Male factor infertility and finally infertility treatment were more prevalent (Table 1). This may also explain why cumulative delivery rates do not differ between women below 30 years and 30–34 years in our cohort.

### Comparison of observational trials on recurrent miscarriages (Table 5)

The association of maternal age and number of preceding miscarriages with further outcome has been addressed in several reviews [8, 9, 36] and recent studies [10, 11, 37, 38]. Since inclusion and exclusion criteria and study designs differ, close comparison of the results is not possible. Nevertheless, evidently our cohort represents a relatively fertile group of women: it comprises women below 40 years of age, and couples who had already undergone in vitro fertilisation or insemination had been excluded. Thus the outcome figures derived are in line with preceding evaluations.

**Table 5:** Comparison of studies on pregnancy and delivery rates after recurrent miscarriages

References	Characteristics of the study			Past history at initial evaluation					Further outcome				
	Design	Women	Reference	Female age (median)	No. of miscarriages (median, range)	Late miscarriages excluded	Secondary miscarriages excluded	Infertility treatment (AIH, IVF)	Period of observation	Pregnancy rate	Deliveries/whole group	Pregnant women achieving deliveries	Delivery rate per age group
Clifford K et al. [10]	Prospective longitudinal	201 Couples with secondary sterility excluded	Next pregnan-cy	34 (22–43)	3 (3–13)	Yes	No	Undefined	Undefined	201 (100 %)	n.a.	138/201 (68.7 %)	<31: 75 % 31–35: 72 % 36–39: 67 % 40: 48 %
Brigham et al. [11]	Prospective longitudinal	325 (of these 23 lost to follow up)	Next pregnan-cy	32 (17–45)	3 (2–10)	Yes	No	Undefined	Undefined	226/302 (74.8 %)	167/302 (55.3 %)	167/226 (73.9 %)	20: 90–88 % 35: 73–68 % 40: 64–58 %
Lund et al. [37]	Follow-up based on Danish Civil Registration System	987	Cumulative	32.7 (20–46)	4 (3–6)	No preclinical m.c. to 22nd week	No	Yes rate undefined	Mean 4 years (2–15 years)	n.a.	665/987 (67.4 %) (after 3rd mc 75 %)	n.a.	20-29: 80 % 30–34:60–70 % 35–39: 60 % 40 +: 45 %
Kaandorp et al. [38]	Additional evaluation Prospective ALIFE-trial	251	Cumulative	34 (22–43)	3 (2–15)	No	No	Yes 13 % of couples	100 weeks + pregnancy outcome	213/251 (84.8 %)	139/251 (55.4 %)	139/213 (65.3 %)	n.a.
Kling et al. [17]	Observational cohort study	228	Cumulative	32 (21–39)	3 (3–7)	Yes preclinical to 15th week	Yes	No	24 months + pregnancy outcome	206/228 (90.4 %)	174/228 (76.4 %)	174/206 (84.5 %)	21–29: 80 % 30–34: 80 % 35–39: 60 %

*n.a.* not assessed, preclinical pregnancy: pregnancy test positive, pelvic ultrasound negative, Secondary miscarriage.: following late miscarriages or delivery, *AIH* allogeneic homologous insemination with the husband’s sperm, *IVF* in vitro fertilisation, *MC* miscarriage

,

Although the outcome after recurrent miscarriages is favourable concerning child birth, the risk of premature delivery and perinatal foetal loss has been observed to be elevated at the same time. In a large retrospective study, the rate of premature delivery was 8.1 %, of premature delivery 2.2 %, and of perinatal death 1.2 % after recurrent miscarriages and thus found to be significantly higher than in a population who had 0-2 miscarriages. Our figures even exceed these numbers which may be explained by the fact that we did not exclude multiple gestation. Moreover, the rate of assisted conception was higher in our cohort (12 of 174, 6.9 vs. 4.2 %) [39]. The figures underline that pregnant women with a history of recurrent miscarriages should be offered special obstetric care. Those who develop subfertility apparently are at higher risk than those who conceive within 6 months after the last miscarriage, irrespective of infertility treatment [40].

In summary, the outcome within 24 months correlates well with basic information derived from the past obstetric history. A preliminary prognostic score could be developed which can help counselling couples individually after a history of primary first trimester recurrent miscarriages (Table 3). There are other factors like andrological infertility, tubal and uterine anomalies, body mass index, and lifestyle which may additionally contribute. Individual fertility factors will possibly remain difficult to recognise in couples conceiving spontaneously, unless further non-invasive markers of embryonic development can be established. In many cases, counselling and emotional support may be the most important element of therapy. Finally, it seems adequate to replace the idea of treatment by measures for preventing a third miscarriage. Main diagnostic and therapeutic options could aim at maintaining fertility as best as possible including both partners.
